# Associations of perchlorate, nitrate, and thiocyanate exposure with arthritis and inflammation indicators in young and middle-aged adults, NHANES 2005-2016

**DOI:** 10.3389/fimmu.2024.1318737

**Published:** 2024-03-01

**Authors:** Hui Zhao, Xuyang Chen, Jianping Ni, Lanlan Fang, Yuting Chen, Yubo Ma, Guoqi Cai, Faming Pan

**Affiliations:** ^1^ Department of Epidemiology and Biostatistics, School of Public Health, Anhui Medical University, Hefei, Anhui, China; ^2^ The Inflammation and Immune Mediated Diseases Laboratory of Anhui Province, Anhui Medical University, Hefei, Anhui, China

**Keywords:** thiocyanate, arthritis, BKMR, inflammation, NHANES

## Abstract

**Background:**

Perchlorates, nitrates, and thiocyanates are prevalent environmental chemicals. Their potential association with arthritis remains unexplored. This study aimed to investigate the link between perchlorate, nitrate, and thiocyanate exposure and arthritis, as well as the potential role of inflammation in this context.

**Methods:**

Utilizing the National Health and Nutrition Examination Survey (NHANES) data spanning from 2005 to 2016, the study enrolled 6597 participants aged 20-59 (young and middle-aged), of which 1045 had arthritis. Employing multivariate logistic regression modeling, multiple linear regression models, restricted cubic spline analysis, Bayesian kernel machine regression (BKMR) modeling, and mediation analysis, we assessed these relationships.

**Results:**

There was a significant positive association between elevated urinary thiocyanate levels and arthritis risk [1.19 (1.11, 1.28)]. This association held true across subgroups of osteoarthritis (OA) [1.24 (1.10, 1.40)] and rheumatoid arthritis (RA) [1.33 (1.15, 1.55)]. Thiocyanate levels displayed a dose-dependent relationship with arthritis risk, showing a linear trend (nonlinear P > 0.05). Conversely, perchlorate and nitrate did not exhibit associations with arthritis risk. BKMR outcomes highlighted a positive correlation between a mixture of perchlorate, nitrate, and thiocyanate and arthritis risk, with thiocyanate being the predominant predictors. Moreover, BKMR and generalized linear model analyses unveiled no significant synergistic effect of urinary perchlorate, nitrate, and thiocyanate on arthritis risk. Furthermore, thiocyanate exposure has been linked to elevated levels of inflammatory indicators (white blood cell, neutrophils, lymphocytes, and systemic immune-inflammatory index (SII)).

**Conclusion:**

Heightened thiocyanate exposure may be linked to elevated arthritis risk, either single or in combined effects. Additionally, thiocyanate exposure is associated with heightened inflammation levels.

## 1 Introduction

Arthritis, characterized by chronic inflammation, holds a significant prevalence. It is estimated that 22.7% (54.4 million) of U.S. adults have received a physician’s diagnosis of various arthritis types (1). Although arthritis encompasses diverse conditions, the prominent ones are osteoarthritis (OA) and rheumatoid arthritis (RA). OA manifests as a gradual degradation of cartilage, coupled with subchondral remodeling and synovial inflammation. In contrast, RA represents a chronic inflammatory and systemic autoimmune disorder. In 2013, arthritis incurred healthcare expenses of $139.8 billion and economic losses of $163.7 billion (2). Besides the substantial financial implications, arthritis constitutes a major contributor to global disability. Approximately 41.7% of U.S (1). individuals with arthritis experience restricted mobility, severely impacting their quality of life. The intricate pathogenesis of arthritis involves a blend of genetic predisposition and environmental triggers (3, 4). Hence, unveiling the underlying factors associated with arthritis holds paramount significance for effective control and prevention strategies.

Perchlorate, nitrate, and thiocyanate are pervasive environmental contaminants with demonstrated wide-ranging health implications (5). Perchlorate finds extensive use in aerospace, fireworks, and explosives, leading to its prevalent presence in drinking water and food sources (6). Nitrates, widely employed in agriculture and food preservation, are commonly found in processed foods (7). Thiocyanate, arising from the reaction of free cyanide with sulfur, occurs in cruciferous vegetables, leafy greens, and dairy products, alongside being a metabolite of cyanide in cigarette smoke (8). Research highlights that urinary levels of these compounds serve as indicators of bodily exposure (9). Perchlorate, nitrate, and thiocyanate are recognized thyroid disruptors, as they competitively hinder thyroid hormone synthesis by binding to the sodium iodide symporter, consequently compromising thyroid function (10). Notably, recent epidemiological investigations have unveiled associations between these substances and the risk of depression, diabetes mellitus (DM), and obesity (11–13). DM and obesity are often concurrent with arthritis. The mechanical impact of excess weight on joints contributes to lower extremity OA, while the instigation of DM-induced arthritis primarily arises from chronic hyperglycemia and insulin resistance, giving rise to oxidative stress and persistent low-grade inflammation (14). Nonetheless, the relationship between exposure to perchlorate, nitrate, and thiocyanate and the risk of arthritis remains ambiguous.

Inflammation is a widely acknowledged underlying contributor to arthritis. Studies has demonstrated that exposure to environmental pollutants, including heightened levels of particulate matter, is correlated with increased CRP levels and a heightened susceptibility to arthritic exacerbations (15). Notably, a population-based study has revealed a plausible association between nitrates and thiocyanates and allergic symptoms in adults. Specifically, nitrates demonstrated a positive correlation with the prevalence of eczema, a condition predominantly characterized by skin inflammation (16). And, nitrates/nitrites facilitate lymphocyte proliferation and cytokine production in humans (17). Furthermore, in chronic inflammatory conditions, appropriate concentrations of thiocyanate mitigate host cell damage induced by the potent oxidant hypochlorous acid (HOCl). However, thiocyanate also promotes the elevation of several pro-inflammatory mediators. Importantly, thiocyanate itself modulates pro-inflammatory gene expression and the release of cytokines/chemokines (18). Animal studies have further elucidated the potential implications of thiocyanate, demonstrating pro-arthritic and pro-inflammatory alterations in thiocyanate-supplemented rats exposed to various arthritis-inducing agents (19). In light of these insights, our conjecture posits that perchlorate, nitrate, and thiocyanate might exert a direct or indirect influence on arthritis, potentially mediated by inflammation. However, the current understanding remains limited concerning whether exposure to chemicals (perchlorate, nitrate, and thiocyanate) corresponds to an augmented arthritis risk and whether such exposure is intertwined with inflammation. Furthermore, the extent of inflammation’s mediating role between these factors remains uncertain. In light of these gaps, we conducted an examination into the potential links between urinary chemicals and arthritis risk. Concomitantly, we endeavored to elucidate whether inflammation operates as a mediating mechanism in this context, thereby offering insights into potential causal pathways.

## 2 Materials and methods

### 2.1 Study design and population

National Health and Nutrition Examination Survey (NHANES) is a population-based epidemiological survey program administered by the Centers for Disease Control and Prevention. The study protocol received ethical clearance from the National Center for Health Statistics, and all study participants provided informed consent. Our study encompassed six survey cycles spanning from 2005 to 2016, each comprising a two-year cycle. Initially, 60,936 participants were included. After excluding individuals older than 60 years (n=11,330), those with missing data on arthritis questionnaire information, inflammation indicators, and urinary levels of perchlorate, nitrate, and thiocyanate (n=42,190), and further eliminating those with missing covariates of interest (n=819), a final cohort of 6,597 participants remained (refer to **Supplementary Figure S1** for the participant screening process). We focused on data from adults aged 20-59 years, as the aging process correlates significantly with arthritis development. Specifically, OA onset relates to an augmented presence of senescent cells within joint tissues (20), and RA patients often exhibit accelerated epigenetic aging (21). Furthermore, a U.S.-based population study revealed a linear decline in age across quartiles of increasing thiocyanate concentration (22). Our findings similarly revealed higher levels of nitrate and thiocyanate among the young and middle-aged cohorts (**Supplementary Table S1**).

Arthritis status was ascertained based on healthcare provider diagnosis. Participants within the 20-59 age range were queried with the questions, “ Has a doctor or other health professional ever told you that you had arthritis?” and “Which type of arthritis was it?”

### 2.2 Measurement of urinary perchlorate, nitrate, thiocyanate and inflammatory indicators

The NHANES Laboratory Protocols webpage presents a comprehensive overview of the applied laboratory methodologies. In brief, urine and blood samples were stored at appropriate temperatures of -20°C or -30°C. To quantify the presence of chemicals in human urine, we employed ion chromatography in conjunction with electrospray tandem mass spectrometry. The process involved chromatographic separation utilizing an IonPac AS16 column with sodium hydroxide as the eluent. Following this separation, the eluate underwent ionization through an electrospray interface, resulting in the generation of negative ions that were then transferred to the mass spectrometer. Analyte concentrations were ascertained through a meticulous process involving the comparison of relative response coefficients against well-established standard concentrations. Employing an automated hematology analyzer, white blood cell, lymphocyte, neutrophil, and platelet counts (expressed as ×10^3^ cells/μL) were determined. The Systemic Immune-inflammation Index (SII) was meticulously computed by (platelet count × neutrophil count)/lymphocyte count. In cases where the concentrations of nitrate, perchlorate, and thiocyanate dipped below the discernible limit of detection (LOD), a standardized protocol was adhered to. Specifically, individuals with levels below this LOD threshold were ascribed values equivalent to the LOD divided by the square root of 2.

### 2.3 Covariates

The covariate selection for this study was informed by our prior research into the interplay between environmental pollutant exposure and arthritis (23, 24). We identified potential confounders and effect modifiers, incorporating them into directed acyclic graphs (DAGs) to inform our modeling strategy, as illustrated in **Supplementary Figure S2**. Chosen covariates encompassed the following confounding factors: sex (male/female), age (continuous), race (Mexican American, other Hispanic, non-Hispanic white, non-Hispanic black, and other race), marital status (categorized as unmarried or separated, married), education (lower than high school, high school or equivalent, and above high school), body mass index (BMI, categorical), drinking(categorical), poverty-to-income ratio (PIR, continuous), diabetes, hypertension, gout, and serum cotinine concentration (continuous), serving as an indicator of exposure to environmental tobacco/secondhand smoke. Furthermore, to mitigate the potential influence of urinary dilution on measurements, urinary creatinine (continuous) was incorporated as a covariate in subsequent analyses (25).

### 2.4 Statistical analysis

All analyses were conducted using R (version 4.2.3) with the ‘rms’, ‘bkmr’, and ‘mediation’ R packages. Initial evaluations of baseline participant characteristics within the arthritic state were conducted using a combination of Chi-square tests, Mann-Whitney U-tests and t-tests. Urinary concentrations of chemicals, namely perchlorate, nitrate, and thiocyanate, were subjected to logarithmic transformation (Ln) to approximate a normal distribution for continuous variables. Additionally, they were categorized into quartiles (Q1-Q4) for analysis as categorical variables. Pearson’s correlation coefficient (r) was harnessed to assess the interrelationships among the log-transformed concentrations of perchlorate, nitrate, and thiocyanate. In parallel, the examination of the connection between these chemicals and the risk of arthritis was undertaken through the application of multivariate logistic regression. Additionally, to investigate the intricate associations between these chemical compounds and indicators of inflammation, the study employed multivariate linear regression models. To assess potential dose-response relationships involving thiocyanate and arthritis, restricted cubic spline (RCS) logistic regression models were applied, using the median as the reference point. All analyses were adjusted for covariates including sex, age, race, marital status, education, BMI, drinking, diabetes, hypertension, gout, PIR, serum cotinine, and urinary creatinine. In addition, subgroup analyses were conducted to investigate potential variations across different arthritis subtypes.

Given the potential for nonlinear and nonadditive dose-response relationships linked to exposure to these chemical mixtures, we employed Bayesian kernel-machine regression (BKMR) modeling for subsequent analyses (26). A Gaussian kernel, implemented through the Markov Chain Monte Carlo (MCMC) algorithm, was used to model the exposure-response function. The MCMC sampler ran for 10,000 iterations. To elucidate the cumulative effect of mixed chemical exposure on arthritis, we incrementally adjusted all chemicals to specific percentiles, with a 5 percentile increase at each step. Furthermore, further insight into the response function of individual chemical exposures concerning arthritis was gained by fixing one chemical at varying percentiles (25th, 50th, and 75th percentiles) while maintaining the other chemicals at median values. Moreover, the utilization of bivariate exposure-response curves allowed us to visually explore the interaction between different chemicals at specific levels of other chemicals. Subsequently, generalized linear models were employed to substantiate the statistical significance of these interactions in relation to arthritis development.

We subsequently conducted a mediation analysis to estimate the potential intermediary influence of inflammatory indicators (white blood cell, lymphocytes, neutrophils, and SII) on the link between thiocyanate (exposure) and arthritis (outcome). The current investigation has harnessed a quasi-Bayesian Monte Carlo technique, employing a total of 1000 simulations, while capitalizing on the utility of a normal approximation. Specifically, the direct effect delineates the influence of thiocyanate exposure on arthritis in isolation from any mediating factors. In contrast, the indirect effect illuminates the repercussion of thiocyanate exposure on arthritis, elucidating the role of intermediary variables in this relationship. To gauge the magnitude of mediation, the proportion of the mediating effect was ascertained by dividing the indirect effect by the total effect.

To enhance the robustness of our findings, we executed various sensitivity analyses. Firstly, acknowledging the intricate sampling structure of NHANES, we conducted survey-weighted multivariate logistic regressions to explore the connection between perchlorate, nitrate, and thiocyanate exposure and arthritis. Secondly, to confirm the stability of the BKMR outcomes, we employed a weighted quantile sum (WQS) regression model. WQS regression offers a reliable method for characterizing environmental mixtures, assessing the collective impact of combined exposures on specific outcomes (27). In our study, we established a WQS index by utilizing quartiles of the urinary chemical mixtures. Forty percent of the total samples served as the test set, while the remaining samples constituted the validation set, subjected to 1000 bootstraps. The final results were interpreted in terms of the effect of a one-quartile increase in the chemical mixture on arthritis. Furthermore, considering the strong association between the aging process and arthritis development, particularly due to the high prevalence of arthritis among the elderly population, we conducted additional analyses to investigate the relationship between thiocyanate exposure and arthritis specifically in older adults.

## 3 Results

### 3.1 Characteristics of participants

Our sample comprised 6,597 participants. Among them, 1,045 (15.8%) received a diagnosis of arthritis (OA 327 (5.6%), RA 224 (3.9%)). Demographic characteristics, as detailed in **Table 1**, illustrated noticeable distinctions between arthritic and non-arthritic participants. Specifically, sex, age, race, marital status, education, BMI, PIR, serum cotinine, diabetes mellitus, hypertension, gout, urinary creatinine, and thiocyanate exhibited significant inter-group variations (*P* < 0.05). **Supplementary Figure S3** presents correlation coefficients for Ln-transformed perchlorate, nitrate, and thiocyanate showcasing their mild interconnections ranging from 0.10 to 0.27. Furthermore, **Supplementary Figure S4** illustrates that thiocyanate concentrations were notably elevated in arthritis and its subtypes (OA, RA) in comparison to control subjects, whereas perchlorate and nitrate levels exhibited no significant differences.

**Table 1:** Basic characteristics of participants by arthritis in U.S. young and middle-aged adults, NHANES 2005–2016.

Characteristics	Participants			*P* value
	Total	Arthritis	Non-arthritis	
Total	6597	1045 (15.8)	5552 (84.2)	
Arthritis subtype				
OA	5879	327 (5.6)	5552 (94.4)	
RA	5776	224 (3.9)	5552 (96.1)	
Other arthritis	6046	494 (8.2)	5552 (91.8)	
Gender				**<0.001**
male	3286 (49.8)	435 (41.6)	2851 (51.4)	
female	3311 (50.2)	610 (58.4)	2701 (48.6)	
Age	39.1 ± 11.3	46.9 ± 9.1	37.7 ± 11.1	**<0.001**
Race				**<0.001**
Mexican American	1176 (17.8)	103 (9.9)	1073 (19.3)	
Other Hispanic	706 (10.7)	101 (9.7)	605 (10.9)	
Non-Hispanic white	2793 (42.3)	587 (56.2)	2206 (39.7)	
Non-Hispanic black	1276 (19.3)	203 (19.4)	1073 (19.3)	
Other race	646 (9.8)	51 (4.9)	595 (10.7)	
Marital status				**<0.001**
Unmarried	2334 (35.4)	244 (23.3)	2090 (37.7)	
Separated	986 (14.9)	262 (25.1)	724 (13.0)	
Married	3277 (49.7)	539 (51.6)	2738 (49.3)	
Education				**<0.001**
Lower than high school	1483 (22.5)	266 (25.4)	1217 (21.9)	
High school or equivalent	1499 (22.7)	266 (25.5)	1233 (22.3)	
Above high school	3615 (54.8)	513 (49.1)	3102 (55.8)	
BMI (kg/m^3^)				**<0.001**
< 25.0	2034 (30.9)	225 (21.6)	1809 (32.7)	
≥ 25.0	4545 (69.1)	817 (78.4)	3728 (67.3)	
Serum cotinine	71.1 ± 140.2	111.3 ± 170.5	63.6 ± 132.4	**<0.001**
Drinking				0.956
Drinker	5027 (76.2)	797 (76.3)	4230 (76.2)	
Non-drinker	1570 (23.8)	248 (23.7)	1322 (23.8)	
Diabetes				**<0.001**
Yes	503 (7.6)	179 (17.1)	324 (5.8)	
No	6094 (92.4)	866 (82.9)	5228 (94.2)	
Hypertension				**<0.001**
Yes	1466 (22.2)	470 (45.0)	996 (17.9)	
No	5131 (77.8)	575 (55.0)	4556 (82.1)	
Gout				**<0.001**
Yes	136 (2.1)	69 (6.6)	67 (1.2)	
No	6461 (97.9)	976 (93.4)	5485 (98.8)	
PIR	2.5 ± 1.7	2.4 ± 1.7	2.6 ± 1.7	**<0.001**
Urinary creatinine	127.0 ± 81.4	121.6 ± 76.2	128.1 ± 82.3	**0.019**
Urine chemicals (ng/mL)				
Perchlorate	3.1 (4.1)	3.3 (4.16)	3.1 (4.0)	0.311
Nitrate	48400.0 (45600.0)	47800.0 (45300.0)	48600.0 (45600.0)	0.294
Thiocyanate	1290.0 (2536.0)	1760.0 (3612.0)	1230.0 (2334.0)	**<0.001**

Normally distributed continuous variables were expressed as mean ± SD; non-normally distributed continuous variables are expressed as median (IQR); Categorical variables were presented as n (%). n, numbers of subjects; %, weighted percentage.

Bold: P<0.05.

### 3.2 Association between perchlorate, nitrate and thiocyanate concentrations and arthritis risk

**Figure 1:**
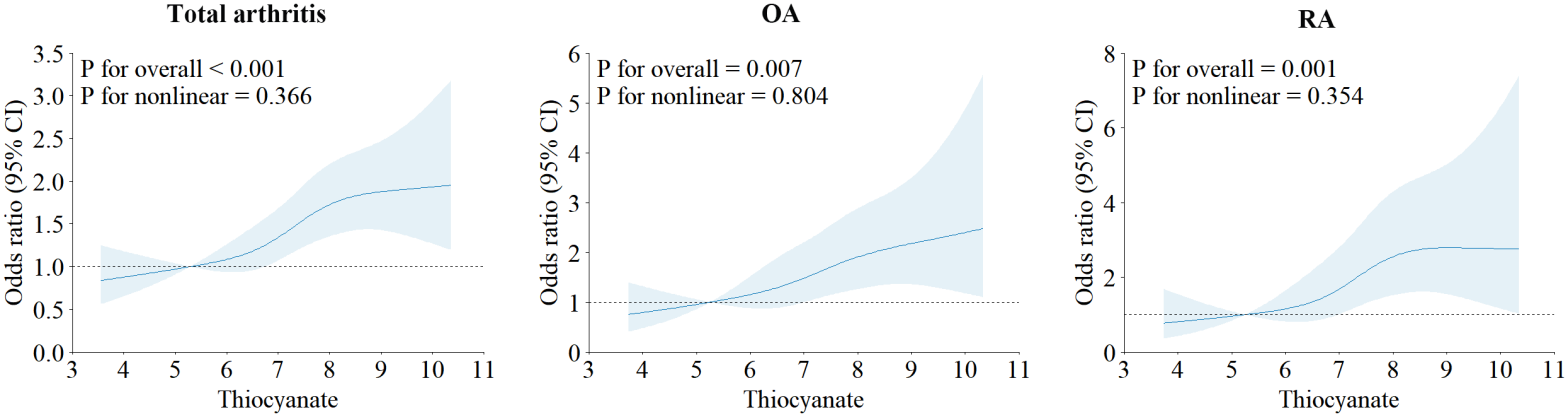
Restricted cubic spline plots of the association between ln-transformed concentration of thiocyanate and arthritis and its subtypes.

**Table 2** presents the outcomes of our multiple logistic regression model, showcasing the associations of perchlorate, nitrate and thiocyanate with arthritis risk. Notably, the highest exposure quartile (Q4) value of thiocyanate (OR 1.60, 95% CI 1.26 to 2.04) demonstrated a significant elevation in arthritis risk compared to Q1 (trend *P* < 0.05). These findings persisted in continuous analyses (*P* < 0.05). Importantly, these associations remained significant when examining the arthritis subtypes, OA (2.03 (1.36, 3.06)), and RA (2.43 (1.52, 3.97)). However, no evident association was observed between perchlorate and nitrate exposure and arthritis risk. To assess the linear association of thiocyanate levels with arthritis risk and its subtypes (OA, RA), we employed a 4-node RCS model. This analysis revealed a linear correlation between thiocyanate and arthritis risk and its subtypes (OA, RA) (all *P* nonlinear > 0.05). Notably, the dose-response connection was also significant (overall *P* < 0.05), as depicted in **Figure 1**.

**Table 2:** Association of perchlorate, nitrate, and thiocyanate with arthritis, NHANES, 2005–2016.

Variables	Continuous	Q1	Q2	Q3	Q4	*P* for trend
	OR (95% CI)	-	OR (95% CI)	OR (95% CI)	OR (95% CI)	
Perchlorate						
Arthritis	1.07 (0.98,1.17)	Ref	1.03 (0.83,1.28)	1.16 (0.93,1.44)	1.23 (0.98,1.55)	0.246
OA	1.03 (0.90,1.19)	Ref	0.90 (0.64,1.26)	1.01 (0.71,1.44)	1.10 (0.76,1.58)	0.717
RA	0.96 (0.81,1.13)	Ref	0.80 (0.52,1.21)	1.07 (0.71,1.63)	0.92 (0.59,1.43)	0.498
Nitrate						
Arthritis	1.01 (0.84,1.22)	Ref	1.11 (0.90,1.37)	1.10 (0.87,1.37)	1.09 (0.84,1.42)	0.790
OA	1.01 (0.84,1.22)	Ref	0.91 (0.64,1.29)	1.04 (0.71,1.51)	0.85 (0.56,1.28)	0.638
RA	1.08 (0.86,1.36)	Ref	0.97 (0.63,1.48)	0.99 (0.63,1.57)	1.04 (0.63,1.71)	0.990
Thiocyanate						
Arthritis	**1.19 (1.11,1.28)**	Ref	1.13 (0.91,1.41)	**1.54 (1.24,1.92)**	**1.60 (1.26,2.04)**	<0.001
OA	**1.24 (1.10,1.40)**	Ref	1.38 (0.93,2.06)	**1.77 (1.21,2.61)**	**2.03 (1.36,3.06)**	0.004
RA	**1.33 (1.15,1.55)**	Ref	1.36 (0.85,2.21)	1.39 (0.87,2.26)	**2.43 (1.52,3.97)**	0.002

Models were adjusted for gender, age, education, serum cotinine, BMI, PIR, race, urinary creatinine, marital status, drinking, hypertension, diabetes and gout. Continuous, ln-transformed concentration of variables; Q, quartile.

Bold: P<0.05.

**Figure 2:**
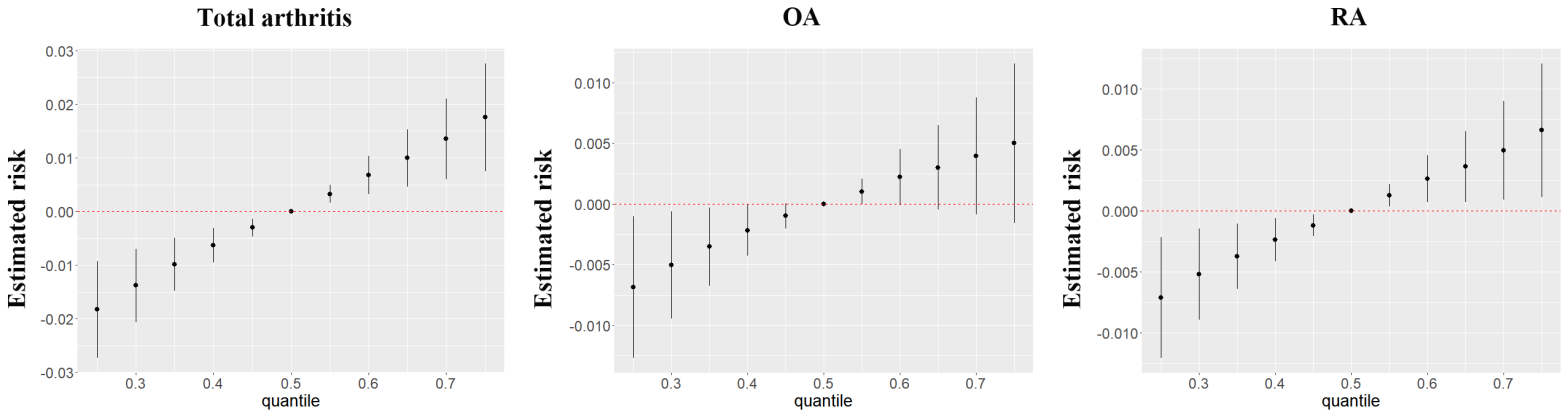
The joint effects of perchlorate, nitrate and thiocyanate mixtures on arthritis and its subtypes were estimated by BKMR models.

Incorporating the BKMR model, our results highlighted a significant positive association between mixed chemical exposure and arthritis risk. Remarkably, within the arthritis subtypes, this relationship was more pronounced in RA but less pronounced in OA (**Figure 2**). The iterative noise plots for both total arthritis and its subtypes demonstrate the stability of the model, as depicted in **Supplementary Figure S5**. Model convergence was achieved after 10,000 iterations, with iterative noise plots displayed for the posterior 50% by default. Noteworthy is the substantial positive effect of thiocyanate on arthritis risk, even when the exposure levels of other chemicals were held at the 25th, 50th, or 75th percentile (**Supplementary Figure S6**). Additionally, our BKMR analyses unveiled a potential interaction between nitrate and thiocyanate in influencing arthritis. Specifically, a descending slope for thiocyanate was noted as nitrate concentrations ascended from the 25th to the 75th percentile (**Supplementary Figure S7**). However, in validation of this interaction, a generalized linear model revealed no statistically significant synergies between the three chemicals (**Supplementary Table S2**).

In the context of sensitivity analyses, survey-weighted multivariate logistic regressions (**Supplementary Table S3**) revealed a positive association between thiocyanate exposure and arthritis risk (1.21 (1.07,1.37)), along with a positive association with the risk of arthritis subtype-OA (1.22 (1.02,1.46)). However, this association dissipated in the case of arthritis subtype-RA (1.20 (0.98,1.48)). Gelman et al (28) argue that in studies focused on examining relationships between exposures and health outcomes, the incorporation of sampling weights may not be requisite. Furthermore, they suggest that additional adjustments in regression analyses for variables used in the computation of sampling weights (e.g., ethnicity, age, and income) could potentially compromise the precision of estimates or even introduce a certain degree of over-adjustment bias. Hence, our earlier results obtained without incorporating sampling weights remain credible, aligning with similar approaches adopted in some prior studies utilizing the NHANES dataset (29, 30). Furthermore, the outcomes of the WQS modeling further reinforced the findings. The collective impact of chemicals mixture exposure on arthritis exhibited consistency with the BKMR results, all indicating a positive association with arthritis risk (arthritis: 1.25 (1.12, 1.41), OA: 1.23 (1.00-1.50), RA: 1.50 (1.10, 1.96)). Remarkably, among the mixed exposure, thiocyanate exerted the most significant influence, as depicted in **Supplementary Figure S8**. In the elderly population, no significant association between thiocyanate exposure and the risk of total arthritis or OA was observed (*P* > 0.05). However, a notable association emerged, indicating that thiocyanate exposure was linked to an increased risk of RA (OR 1.15, 95%CI 1.00-1.32) (**Supplementary Table S4**).

### 3.3 Associations between perchlorate, nitrate, thiocyanate and inflammation indicators and mediation analyses

We investigated the relationships between exposure to chemicals and inflammatory indicators using multiple linear regression models. Our findings revealed a positive association between thiocyanate exposure and indicators of inflammation, including white blood cell (β 0.21, 95% CI 0.15-0.26), neutrophils (β 0.17, 95% CI 0.13-0.21), lymphocytes (β 0.03, 95% CI 0.01-0.05), and SII (β 13.19, 95% CI 5.37-21.00) (**Table 3**). It is noteworthy that this study observed an association between low-dose (Q2) perchlorate exposure and reduced lymphocyte counts, displaying a linear trend in quartile-based results. Otherwise, the analysis did not reveal any noteworthy connection between exposure to perchlorate or nitrate and the measured inflammatory indicators.

**Table 3:** Multivariate linear regression of ln-transformed perchlorate, nitrate and thiocyanate levels with inflammatory indicators.

Variables	Continuous	Q1	Q2	Q3	Q4	*P* for trend
	β (95% CI)	-	β (95% CI)	β (95% CI)	β (95% CI)	
Perchlorate						
White blood cell^#^	-0.01(-0.07,0.05)	Ref	-0.08(-0.22,0.07)	-0.03(-0.19,0.12)	0.02(-0.14,0.18)	0.557
Neutrophils^#^	-0.02(-0.07,2.81)	Ref	-0.03(-0.15,0.09)	0.01(-0.12,0.13)	-0.00(-0.13,0.13)	0.935
Lymphocyte^#^	0.01(-0.01,0.02)	Ref	**-0.05(-0.09,-0.00)**	-0.04(-0.09,0.01)	0.02(-0.04,0.07)	0.014
SII	-3.19(-12.32,5.93)	Ref	-5.90(-27.94,16.14)	1.74(-21.39,24.86)	-3.30(-27.41,20.80)	0.901
Nitrate						
White blood cell^#^	0.04(-0.04,0.13)	Ref	0.00(-0.15,0.15)	0.03(-0.13,0.20)	0.09(-0.09,0.27)	0.704
Neutrophils^#^	0.05(-0.03,0.12)	Ref	0.01(-0.11,0.13)	0.04(-0.09,0.18)	0.09(-0.06,0.24)	0.654
Lymphocyte^#^	-0.02(-0.05,0.01)	Ref	-0.01(-0.06,0.03)	-0.03(-0.09,0.02)	-0.03(-0.08,0.03)	0.681
SII	9.93(-3.18,23.04)	Ref	17.68(-4.72,40.08)	11.08(-13.84,35.99)	13.39(-14.51,41.29)	0.492
Thiocyanate						
White blood cell^#^	**0.21(0.15,0.26)**	Ref	-0.01(-0.14,0.14)	**0.18(0.03,0.33)**	**0.68(0.51,0.85)**	<0.001
Neutrophils^#^	**0.17(0.13,0.21)**	Ref	0.03(-0.08,0.15)	**0.21(0.09,0.33)**	**0.53(0.39,0.67)**	<0.001
Lymphocyte^#^	**0.03(0.01,0.05)**	Ref	-0.01(-0.06,0.03)	-0.01(-0.06,0.04)	**0.12(0.06,0.17)**	<0.001
SII	**13.19(5.37,21.00)**	Ref	8.34(-13.53,30.21)	**38.83(16.19,61.48)**	**37.80(11.86,63.74)**	0.001

Models were adjusted for gender, age, education, serum cotinine, BMI, PIR, race, urinary creatinine, marital status, drinking, hypertension, diabetes and gout. SII, systemic immune-inflammation index.

#: 1000 cells/μL, Bold: P<0.05.

Moreover, we conducted mediation analyses to evaluate the potential role of inflammatory indicators in mediating the connection between thiocyanate and arthritis risk. After accounting for potential confounders (**Table 4**), the results demonstrated that SII exhibited a noteworthy mediating effect on the relationship between thiocyanate and arthritis risk, with a mediation ratio of 2.19% (P < 0.05). Nonetheless, the analysis did not reveal any notable mediation effects for white blood cell, neutrophils, or lymphocytes.

**Table 4:** The mediating effects of inflammatory indicators on the association between ln-transformed thiocyanate and risk of arthritis.

Variable	Indirect effects	Direct effects	Total effects	Mediated proportion (%)	*P*-value
	β (95%CI)	β (95%CI)	β (95%CI)		
White blood cell	6.07e-04(-2.29e-04,0.00)	0.018(0.009,0.03)*	0.018(0.010,0.03)*	–	0.160
Neutrophils cell	8.38e-04(-7.71e-06,0.00)	1.73e-02(8.51e-03,0.03)*	1.81e-02(9.46e-03,0.03)*	–	0.068
Lymphocyte cell	-1.17e-04(-6.33e-04,0.00)	0.018(0.009,0.03)*	0.018(0.009,0.03)*	–	0.620
SII	4.24e-04(2.86e-05,0.00)*	1.80e-02(9.09e-03,0.03)*	1.84e-02(9.57e-03,0.03)*	**2.19%**	0.028

Models were adjusted for gender, age, education, serum cotinine, BMI, PIR, race, urinary creatinine, marital status, drinking, hypertension, diabetes and gout. SII, systemic immune-inflammation index.

*P < 0.05, Bold: P<0.05.

## 4 Discussion

In this study, our investigation delved into the potential connection between urinary perchlorate, nitrate, and thiocyanate levels and the vulnerability to arthritis in young and middle-aged adults. The findings of this study have unveiled two significant and original revelations. Firstly, the models examining individual and combined exposure consistently underscored thiocyanate’s robust association with arthritis risk, with this association displaying a linear trend. Furthermore, our analysis revealed that elevated thiocyanate exposure correlated with increased levels of inflammatory indicators.

Dietary sources encompassing thiocyanate comprise almonds, cabbage, kale, broccoli, cassava, and other cruciferous vegetables. Furthermore, a pivotal contributor to thiocyanate exposure emerges from tobacco or secondhand smoke. This stems from the enzymatic conversion of cigarette smoke-derived cyanide into thiocyanate, followed by the subsequent conversion to cyanate catalyzed by myeloperoxidase (MPO) (31). Prior *in vitro* studies have highlighted how thiocyanate ions (SCN-) counteract the accumulation of potentially hazardous hydrogen peroxide (H_2_O_2_) and hypochlorite (OCl-), mitigating the cytotoxicity imparted by MPO released from white blood cell (32). Additionally, thiocyanates have demonstrated efficacy in alleviating chronic airway inflammation and stubborn bacterial infections by curbing neutrophil infiltration and inhibiting glutathione sulfonamide in a murine cystic fibrosis model (33). Nonetheless, it is imperative to acknowledge the existence of a dose-response dynamic regulating the interaction between thiocyanate (OSCN-) and inflammation. Specifically, low doses of OSCN- activate the NF-κB pathway in airway epithelial cells via protein kinase A (PKA), while elevated doses lead to cellular necrosis, thereby releasing IL-33 and instigating inflammation (34). Remarkably, our study similarly discerned a marked dose-response correlation between thiocyanate exposure and inflammatory indicators, including white blood cells, neutrophils, lymphocytes, and SII (all trends *P*<0.05).

Recent investigations have underscored the direct detrimental impact of cyanate on vascular tissue through the generation of heightened levels of reactive oxygen species, and cyanate has been identified as an inducer of oxidative stress impairment and lipid accumulation by inhibiting the Nrf2/HO-1 pathway (35). Epidemiological inquiries have established a connection between thiocyanate exposure and the provocation of allergic inflammation, correlating positively with the prevalence of allergy-associated symptoms (16). Additionally, heightened levels of SCN- can perturb neutrophil activation, contributing to an imbalanced neutrophil function within smokers and thereby compromising the innate immune response (36). Notably, the significance of oxidative stress and inflammatory response mechanisms in arthritic disease development is well-recognized (37). Collectively, these insights tentatively imply a plausible linkage between thiocyanates and susceptibility to arthritis.

In our study, we observed a significant dose-response relationship between individual thiocyanate exposure and the risk of arthritis, including its subtypes (OA and RA). Furthermore, the results from the BKMR model indicated that combined exposure to chemicals was positively associated with arthritis risk, with thiocyanate being the primary influencing factor. While epidemiological studies on thiocyanate and arthritis risk are limited, animal studies have provided supportive evidence (19, 38). These studies indicate that exposure to thiocyanate heightened inflammatory responses to various factors known to trigger arthritic and fibrotic inflammation. Moreover, when administered in isolation, thiocyanate induced systemic arthritic symptoms in rats. Additionally, it significantly exacerbated the severity of mycobacterial arthritis (MIA), collagen-induced arthritis (CIA), and descending phytolithane-induced arthritis (PIA), acting as a facilitator of pathological processes. It is noteworthy that while thiocyanate is generally considered relatively benign, this may not hold true for rats or humans experiencing immunoinflammatory stress alongside concurrent leukocyte activation (19).

SII, as an indicator of systemic inflammatory response, has demonstrated prognostic value in patients with tumors (39). In recent years, a growing body of evidence has substantiated the efficacy of the SII in evaluating the severity and advancement of psoriatic arthritis (PsA) (40). Among individuals exhibiting elevated SII levels, a persistent state of immune system activation prevails, thereby fostering chronic inflammation not only within the joints but also affecting other bodily tissues (41, 42). Findings from a case-control study underscored significantly heightened SII levels in RA patients compared to their healthy counterparts, with SII demonstrating a correlation with disease severity (43). Moreover, a recent population-based investigation has unveiled a positive correlation between SII and RA risk, potentially serving as a predictive marker for RA risk among adults in US (44). Our study revealed a mediating effect of only 2.19% (P < 0.05) for the SII in the relationship between thiocyanate exposure and arthritis risk. However, it is crucial to acknowledge that this modest mediation percentage might be attributable to residual biases, including unmeasured variables. Additionally, potential batch effects in sample measurements could influence these findings. Therefore, caution is warranted when interpreting this mediation outcome. The limited mediating role of SII in the association between thiocyanate exposure and arthritis risk necessitates further research for validation.

Our study boasts several noteworthy advantages. Firstly, it represents the inaugural investigation into the connection between perchlorate, nitrate, thiocyanate, and arthritis within the context of a substantial sample size. Secondly, we have, for the first time, elucidated a significant positive correlation between thiocyanate exposure and inflammatory indicators (white blood cell, centrocytes, lymphocytes, and SII) within a population. Furthermore, we adopted both BKMR and WQS models to comprehensively evaluate the impact of combined perchlorate, nitrate, and thiocyanate exposures on arthritis, thereby enhancing the robustness of our findings. Lastly, our study harnessed a wealth of sample data and comprehensive covariate information, thereby fortifying the accuracy and reliability of the conclusions derived from this investigation.

Nonetheless, our study does present certain limitations. Firstly, the identification of arthritis and its subtypes in our study relied on questionnaires and self-reports, methods that are susceptible to bias. Secondly, despite our efforts to control for numerous confounding factors, the absence of certain potential confounders, such as cruciferous vegetable intake and medication usage, due to limitations in the NHANES dataset, may have led to an overestimation of the impacts of thiocyanate exposure. Thirdly, the absence of documentation for some typical inflammatory factors (e.g., TNF-α, interleukin-6, interleukin-10, etc.) in the NHANES data meant that these relevant metrics could not be included, potentially limiting the comprehensiveness of our results. Lastly, the cross-sectional design of our study restricts our ability to infer causality and temporality in association and mediation analyses. Thus, while our findings suggest potential mediating relationships, causality should be interpreted cautiously. Nevertheless, we anticipate that our study contributes valuable insights into the association between thiocyanate exposure, inflammation, and arthritis.

## 5 Conclusions

In conclusion, our study establishes a significant and positive association between thiocyanate exposure and the risk of developing arthritis, alongside a similar positive correlation with inflammatory markers (white blood cell, centrocytes, lymphocytes, and SII) in young and middle-aged adults. Moreover, we identify SII as a mediator between thiocyanate and arthritis risk. Nevertheless, to ascertain the reliability and establish causal relationships for these findings, further prospective and mechanistic studies are warranted.

## Data availability statement

Publicly available datasets were analyzed in this study. This data can be found here: https://www.cdc.gov/nchs/nhanes/index.htm.

## Ethics statement

The studies involving humans were approved by National Center for Health Statistics (NCHS) Ethics Review Board/Centers for Disease Control and Prevention. The studies were conducted in accordance with the local legislation and institutional requirements. The participants provided their written informed consent to participate in this study. Ethical review and approval were waived for this study as it solely used publicly available data for research and publication.

## Author contributions

HZ: Conceptualization, Methodology, Software, Visualization, Writing – original draft, Writing – review & editing. XC: Conceptualization, Methodology, Software, Visualization, Writing – original draft. JN: Conceptualization, Methodology, Software, Visualization, Writing – original draft. LF: Data curation, Software, Visualization, Writing – review & editing. YC: Investigation, Software, Visualization, Writing – review & editing. YM: Software, Visualization, Writing – review & editing. GC: Software, Visualization, Writing – review & editing. FP: Funding acquisition, Project administration, Supervision, Writing – review & editing.
