# Supplementary material for: Associations of perchlorate, nitrate, and thiocyanate exposure with arthritis and inflammation indicators in young and middle-aged adults, NHANES 2005-2016
Source: Front Immunol. 2024 Mar 1;15:1318737. doi: 10.3389/fimmu.2024.1318737 (PMC10940346; doi:10.3389/fimmu.2024.1318737)
Supplement: Supplementary file 1 [file DataSheet_1.docx]

**Supplementary material**

**Associations of perchlorate, nitrate, and thiocyanate exposure with arthritis and inflammation indicators in young and middle-aged adults, NHANES 2005-2016**

Hui Zhao^1,2^†, Xuyang Chen^1,2^†, Jianping Ni^1,2^†, Lanlan Fang^1,2^, Yuting Chen^1,2^, Yubo Ma^1,2^, Guoqi Cai^1,2^, Faming Pan^1,2^*

1 Department of Epidemiology and Biostatistics, School of Public Health, Anhui Medical University, Hefei, Anhui, China;

2 The Inflammation and Immune Mediated Diseases Laboratory of Anhui Province, Anhui Medical University, 81 Meishan Road, Hefei, Anhui, 230032, China;

***Correspondence:** Faming Pan, PhD, Department of Epidemiology and Biostatistics, School of Public Health, Anhui Medical University, 81 Meishan Rd, Hefei, Anhui 230032, China;

E-mail: famingpan@ahmu.edu.cn.

† Hui Zhao, Xuyang Chen and Jianping Ni contributed equally to this work and should be considered co-first authors.

**Fig. S1** Participant flowchart, NHANES 2005–2016


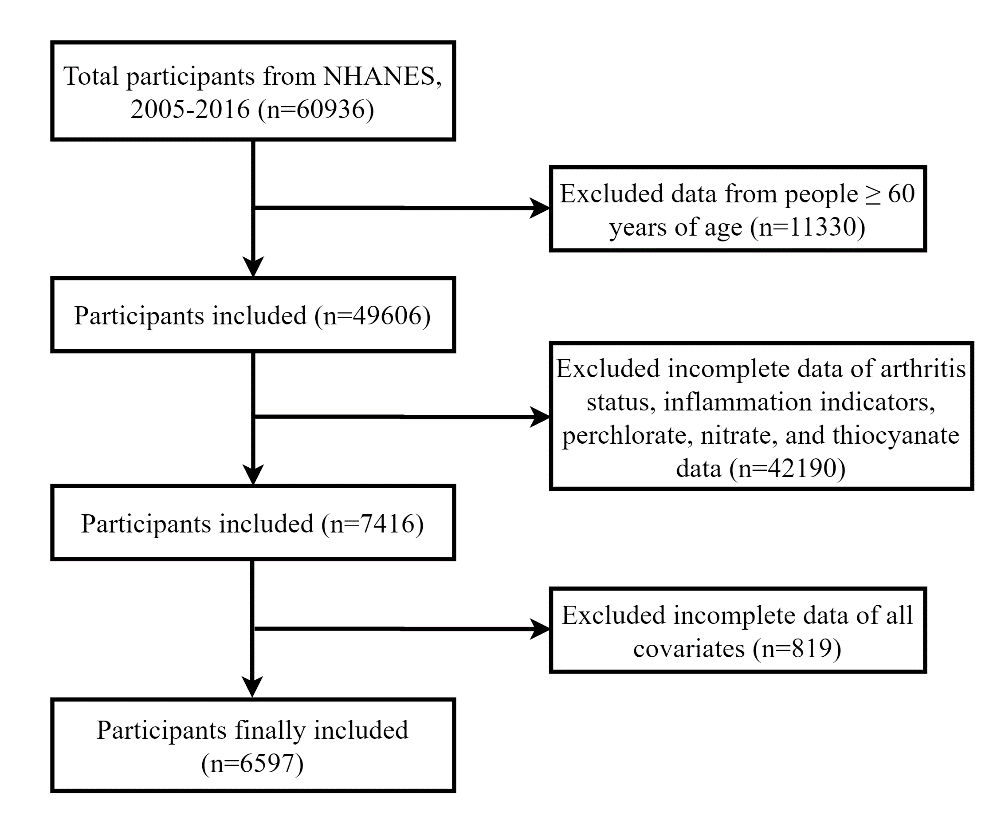


**Fig. S2** Directed Acyclic Graph (DAG) of the association between perchlorate, nitrate, and thiocyanate and arthritis


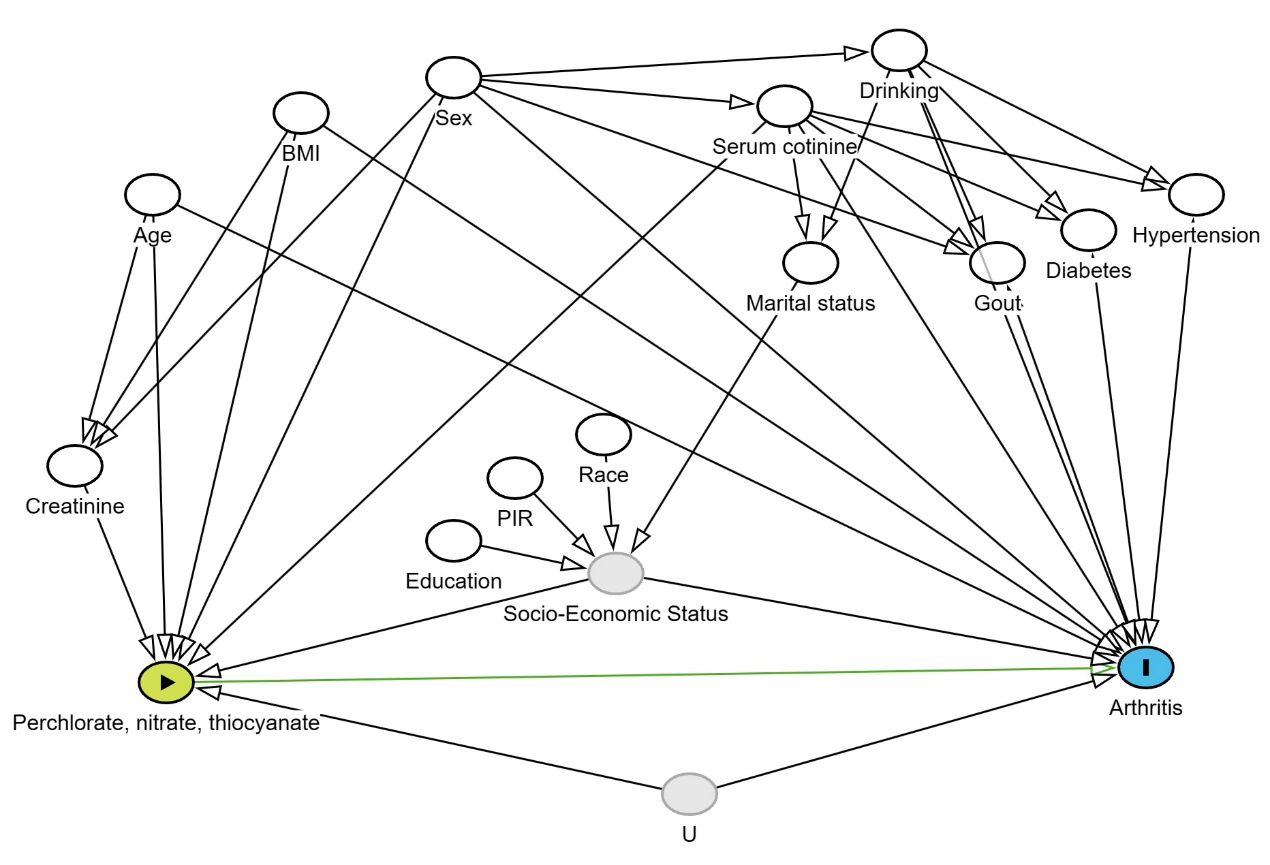


Black circles represent ancestors of the exposure and outcome (ie, confounders), and gray circles represent unobserved (ie, latent) variables. Green lines represent causal paths, and black lines represent biasing paths. U, unmeasured confounders.

**Fig. S3** Pearson’s correlation matrix among ln-transformed chemicals.


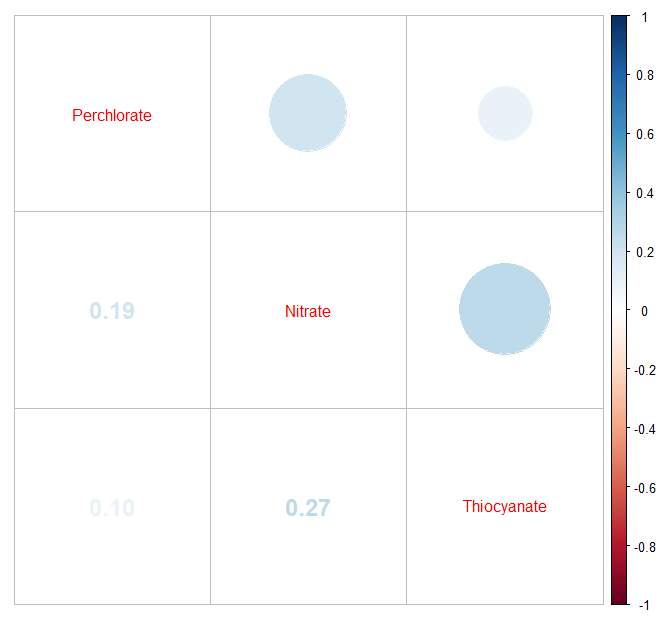


**Fig. S4** Assessing perchlorate, nitrate, and thiocyanate concentrations in total arthritis and its subtypes following ln-transformed.


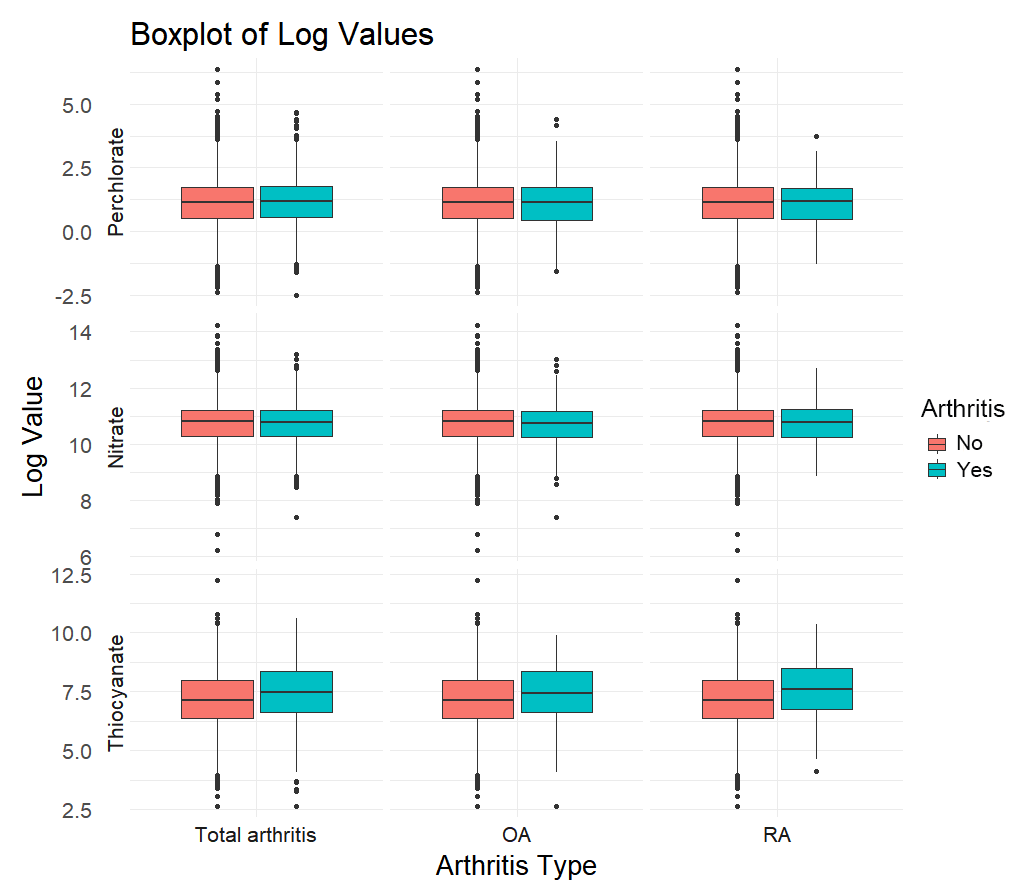


**Fig. S5** Iteration noise plots for arthritis and its subtypes in the BKMR model


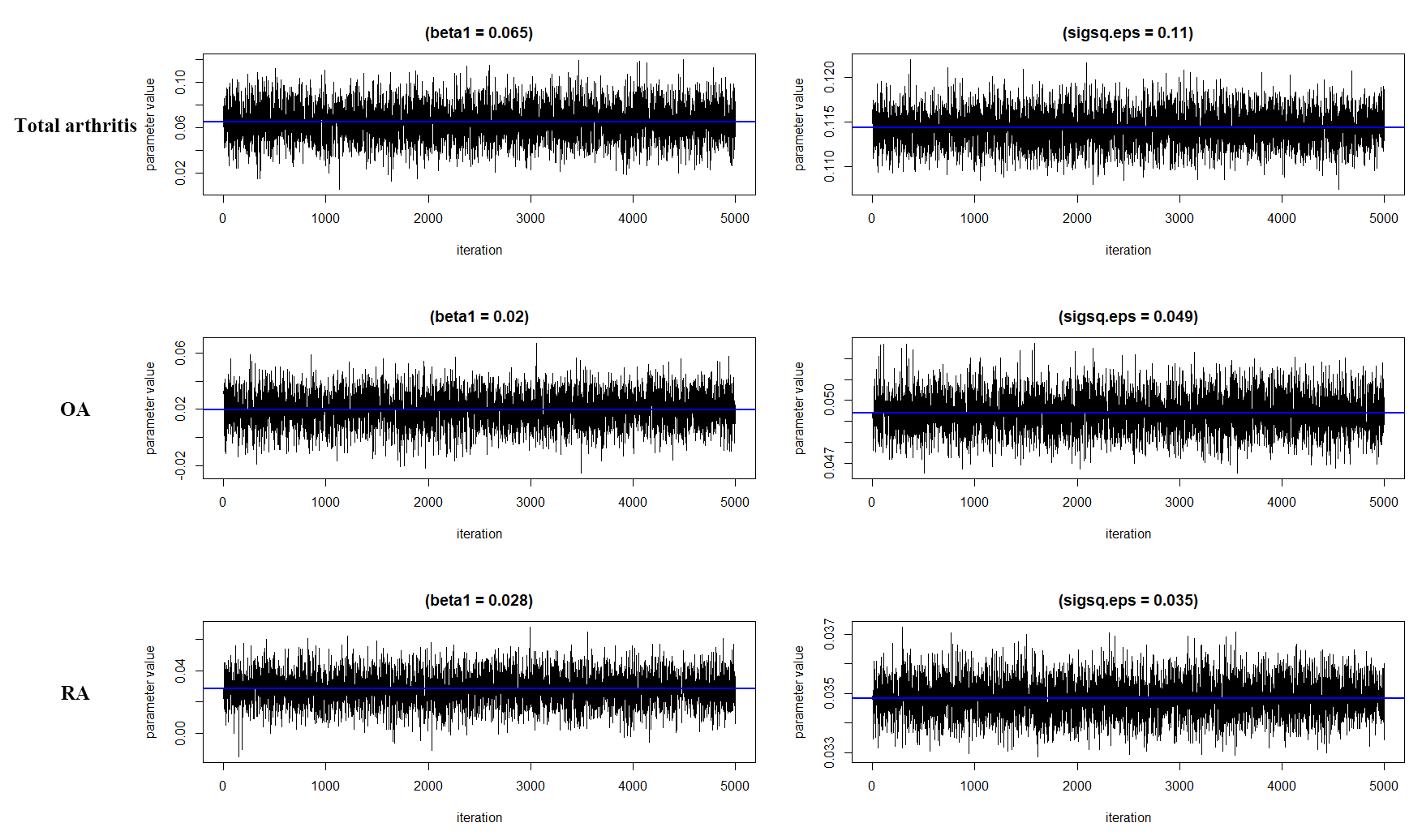


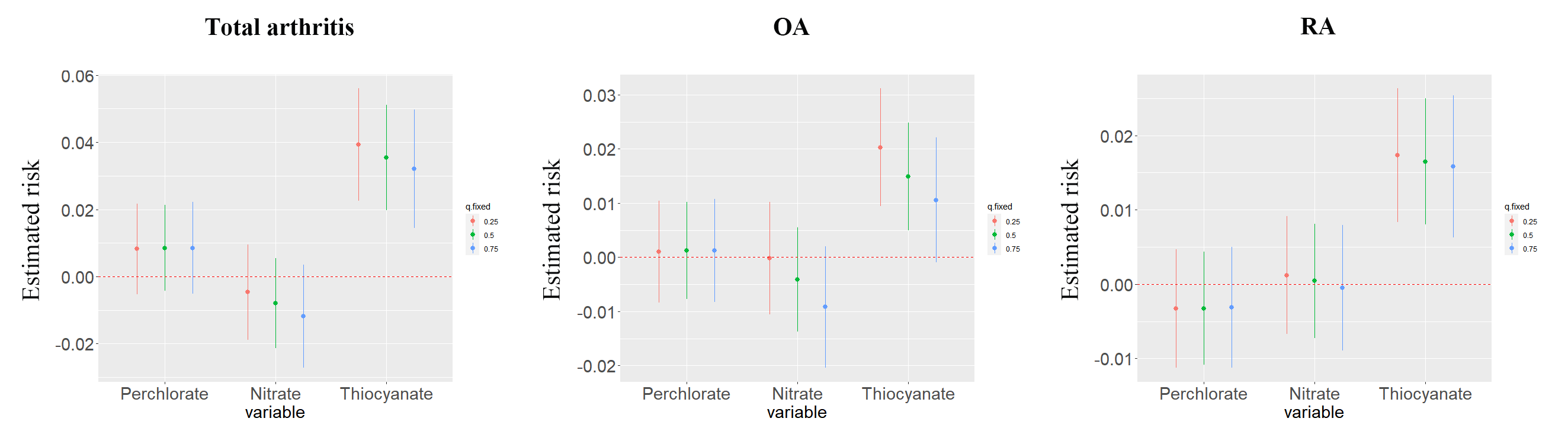
**Fig. S6** Associations of perchlorate, nitrate, thiocyanate with arthritis risk were estimated by BKMR models in total and its subtypes, when other chemicals were held at their corresponding 25th (red), 50th (green) or 75th (blue) percentile, respectively.

**Fig. S7** The interaction of perchlorate, nitrate, thiocyanate on arthritis and its subtypes.


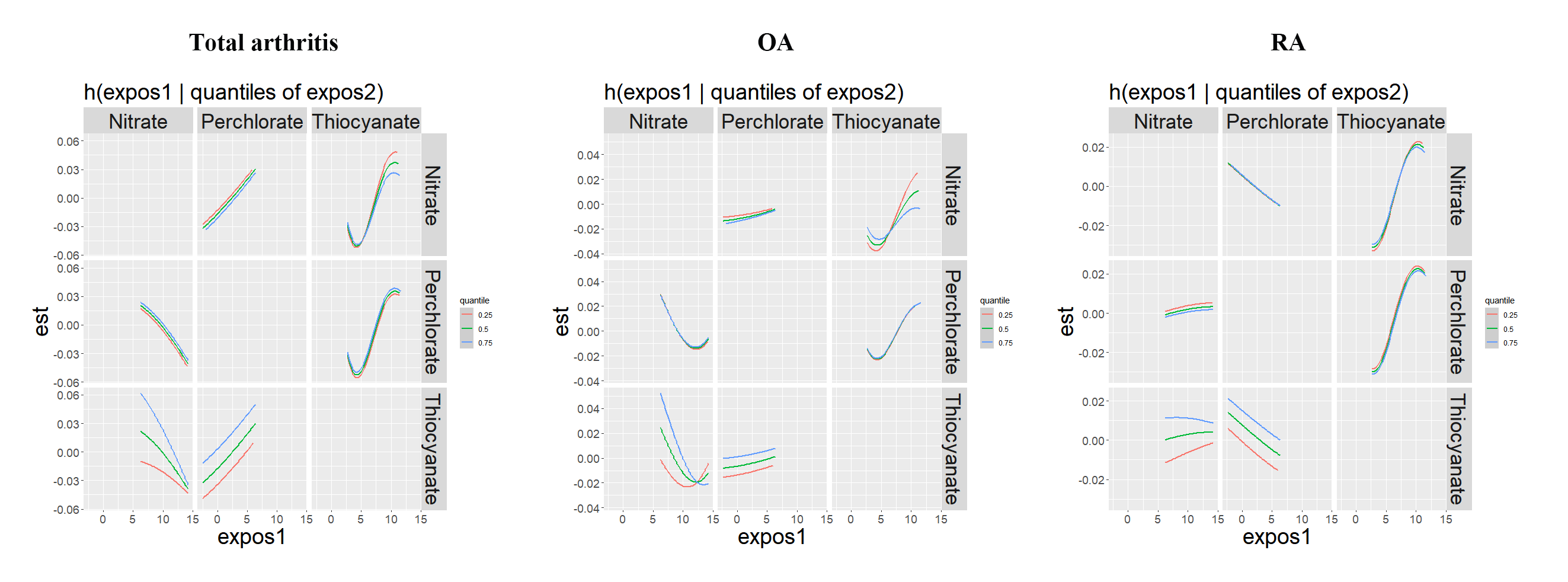


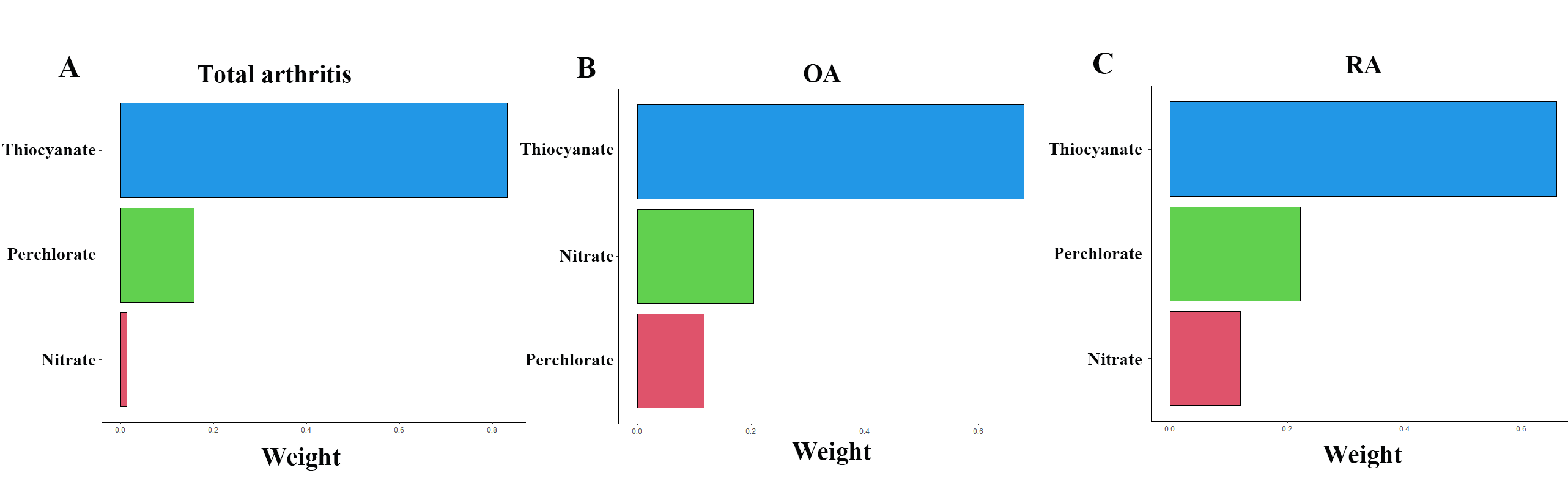
**Fig. S8** Weighted values of perchlorate, nitrate and thiocyanate for arthritis and its subtypes in the WQS model.

**Table S1** Evaluation of disparities in perchlorate, nitrate and thiocyanate concentrations in young and middle-aged and elderly populations

| Variables | 20-59 years  (n = 6597) | > 60 years  (n = 3217) | P-value |
| --- | --- | --- | --- |
| Perchlorate | 3.13 (4.06) | 3.10 (3.76) | 0.344 |
| Nitrate | 48400 (45600) | 36700 (36100) | **< 0.001** |
| Thiocyanate | 1290 (2540) | 804 (1208) | **< 0.001** |

Non-normally distributed continuous variables are expressed as median (IQR).

**Table S2** Interactions between perchlorate, nitrate, thiocyanate in association with arthritis: generalized linear models

| Interaction variable | OR (95% CI) | z | *P* |
| --- | --- | --- | --- |
| Total arthritis |  |  |  |
| Perchlorate * Nitrate | 1.00 (0.91,1.09) | -0.04 | 0.969 |
| Perchlorate * Thiocyanate | 0.99 (0.93,1.05) | -0.29 | 0.774 |
| Nitrate * Thiocyanate | 0.97 (0.90,1.04) | -0.93 | 0.353 |
| OA |  |  |  |
| Perchlorate * Nitrate | 1.04 (0.90,1.19) | 0.53 | 0.597 |
| Perchlorate * Thiocyanate | 0.97 (0.87,1.07) | -0.69 | 0.493 |
| Nitrate * Thiocyanate | 0.93 (0.82,1.04) | -1.25 | 0.211 |
| RA |  |  |  |
| Perchlorate * Nitrate | 0.94 (0.78,1.12) | -0.67 | 0.505 |
| Perchlorate * Thiocyanate | 1.00 (0.88,1.12) | -0.06 | 0.954 |
| Nitrate * Thiocyanate | 0.93 (0.80,1.08) | -0.91 | 0.364 |

Models were adjusted for gender, age, education, serum cotinine, BMI, PIR, race, urinary creatinine, marital status, drinking, hypertension, diabetes and gout.

**Table S3** Association of perchlorate, nitrate, thiocyanate with arthritis and its subtypes: survey-weighted multivariate logistic regression

| variables | Continuous | Q1 | Q2 | Q3 | Q4 | *P* trend |
| --- | --- | --- | --- | --- | --- | --- |
|  | OR (95%CI) |  | OR (95%CI) | OR (95%CI) | OR (95%CI) |  |
| Perchlorate |  |  |  |  |  |  |
| Arthritis | 1.03 (0.88,1.21) | Ref | 0.83 (0.57,1.20) | 0.98 (0.66,1.45) | 1.07 (0.72,1.59) | 0.570 |
| OA | 1.03 (0.800,1.32) | Ref | 0.76 (0.43,1.34) | 0.79 (0.43,1.44) | 1.00 (0.55,1.81) | 0.620 |
| RA | 0.85 (0.62,1.15) | Ref | 0.72 (0.34,1.52) | 0.91 (0.43,1.94) | 0.82 (0.42,1.62) | 0.840 |
| Nitrate |  |  |  |  |  |  |
| Arthritis | 1.02 (0.84,1.23) | Ref | 0.99 (0.68,1.45) | 1.26 (0.84,1.90) | 0.92 (0.57,1.47) | 0.350 |
| OA | 1.05 (0.80,1.36) | Ref | 1.14 (0.64,2.02) | 1.56 (0.90,2.73) | 0.98 (0.51,1.92) | 0.260 |
| RA | 1.04 (0.76,1.44) | Ref | 0.62 (0.30,1.29) | 0.87 (0.40,1.90) | 0.87 (0.39,1.91) | 0.600 |
| Thiocyanate |  |  |  |  |  |  |
| Arthritis | **1.21 (1.07,1.37)** | Ref | 1.26 (0.84,1.89) | **1.72 (1.15,2.56)** | **1.93 (1.28,2.92)** | 0.008 |
| OA | **1.22 (1.02,1.46)** | Ref | 1.59 (0.87,2.92) | **2.11 (1.17,3.79)** | **2.13 (1.18,3.85)** | 0.047 |
| RA | 1.20 (0.98,1.48) | Ref | 0.82 (0.47,1.45) | 1.36(0.76,2.45) | 1.49 (0.82,2.71) | 0.170 |

The model adjusted for gender, age, education, serum cotinine, BMI, PIR, race, urinary creatinine, marital status, drinking, hypertension, diabetes and gout. Continuous, ln-transformed concentration of variables.

**Bold:** *P*<0.05

**Table S4** Association of thiocyanate with arthritis in older adults, NHANES, 2005–2016.

| Variables | Participants | | Continuous | Q1 | Q2 | Q3 | Q4 | *P* for  trend |
| --- | --- | --- | --- | --- | --- | --- | --- | --- |
|  | Arthritis | Non-arthritis | OR (95% CI) | - | OR (95% CI) | OR (95% CI) | OR (95% CI) |  |
| Total arthritis | 1517 | 1495 | 1.01 (0.94,1.09) | Ref | 1.06 (0.86,1.31) | 1.06 (0.85,1.31) | 1.07 (0.86,1.34) | 0.923 |
| OA | 615 | 1495 | 0.93 (0.84,1.03) | Ref | 0.94 (0.71,1.23) | 0.96 (0.73,1.26) | 0.82 (0.61,1.12) | 0.657 |
| RA | 270 | 1495 | **1.15 (1.00,1.32)** | Ref | 1.45 (0.98,2.17) | 1.30 (0.87,1.96) | 1.51 (1.00,2.29) | 0.200 |

Models were adjusted for gender, age, education, serum cotinine, BMI, PIR, race, urinary creatinine, marital status, drinking, hypertension, diabetes and gout. Continuous, ln-transformed concentration of variables; Q, quartile.

**Bold:** *P*<0.05
